# Towards a values framework for integrated health services: an international Delphi study

**DOI:** 10.1186/s12913-020-5008-y

**Published:** 2020-03-18

**Authors:** Nick Zonneveld, Jörg Raab, Mirella M. N. Minkman

**Affiliations:** 1 TIAS School for Business and Society/Tilburg University, The Netherlands Warandelaan 2, 5037 AB Tilburg, The Netherlands; 2 Vilans, National Centre of Excellence in Long Term Care, The Netherlands Catharijnesingel 47, 3511 GC Utrecht, The Netherlands; 3 Department of Organization Studies, Tilburg University, The Netherlands Warandelaan 2, 5037 AB Tilburg, The Netherlands

**Keywords:** Integrated health services delivery, Values, Normative integration, Governance, Integrated care, Framework, Model

## Abstract

**Background:**

In order to organize person-centered health services for a growing number of people with multiple complex health and social care needs, a shift from fragmented to integrated health services delivery has to take place. For the organization of governance in integrated health services, it is important to better understand the underlying factors that drive collaboration, decision-making and behavior between individuals and organizations. Therefore, this article focuses on these underlying normative aspects of integrated health services. This study investigates the values that underpin integrated health services delivery as a concept, by examining the extent to which an initial literature based set of underlying values underpins integrated care and the relevance of these values on the different levels of integration.

**Methods:**

An international Delphi study with 33 experts from 13 different countries was carried out to examine the initial set of underlying values of integrated health services. In addition, the relevance of the values was assessed on the different levels of integration: personal level, professional level, management level and system level.

**Results:**

The study resulted in a refined set of 18 values of integrated health services developed in three Delphi study rounds. In addition, the results provided insight into the relevance of these values on the personal level (e.g. ‘trustful’), professional level (e.g. ‘collaborative’), management level (e.g. ‘efficient’) and system level (e.g. ‘comprehensive’) of integration. Some of the values score consistent across the different levels of integration while other values score inconsistent across these levels.

**Conclusions:**

The Delphi study resulted in an international normative basis for integrated health services delivery as a concept. The values can be used as ingredients for a values framework and provide a better understanding of the normative aspects of integrated health services delivery. Future research could focus on associated behaviors in practice, the relationship between normative integration and governance, and differences between the value priorities of stakeholder groups.

## Background

Health systems are facing the challenges of aging populations and a growing number of people with multiple chronic conditions [1, 2]. An increasing number of people develops multiple complex health and social care needs, which require various types of services that transcend traditional sectors like primary care, long-term care and social care [3]. This implies that actors and services have to be connected, coordinated and organized around a person [4, 5]. However, fragmentation of health services is still a frequently encountered problem in many countries [6–8]. Therefore, it is widely acknowledged that a shift towards integrated health services delivery has to take place [9–12]. Integrated health services delivery is defined as “an approach to strengthen people-centred health systems through the promotion of the comprehensive delivery of quality services across the life-course, designed according to the multidimensional needs of the population and the individual and delivered by a coordinated multidisciplinary team of providers working across settings and levels of care.” [12], (p., 10).

While widely applied and under development in many countries, integrated health services delivery is often a complex and non-hierarchical undertaking with various implications [13–16]. In addition to the implementation of interventions, integration requires changes in healthcare workforce, behavior, organizational design, governance and funding on multiple organizational levels [17–20]. Furthermore, as integrated health services delivery is a collective process, collaboration is needed between actors e.g. service users, informal carers, various care professionals and care providers, governments and health insurers. Although they are often interdependent and subsequently collaborate, at the same time these actors often have different institutional constraints, interests, professional backgrounds, views and objectives. This complicates the alignment of the collaboration processes [21]. Since integrated health services delivery often takes place in collaborative networks in the absence of a formal hierarchy, traditional top-down governance within organizations is not always suitable or effective [4]. Therefore, a shift towards less hierarchical network governance, focusing on collaborative relationships between individuals and organizations, seems more appropriate [22, 23]. This type of governance is known as collaborative or shared governance, implying that networks are jointly and horizontally governed by the interacting organizations in the network [24, 25].

To effectively organize shared governance in integrated health services delivery, it is important to be aware of the circumstances in the network. Provan and Kenis (2008) outline four critical contingencies for effective shared governance: 1) trust has to be widely shared among the network (high-density, decentralized trust), 2) there are relatively few network actors, 3) there is a high goal consensus and 4) there is a low need for network-level competencies [25]. To understand shared governance and collaborative processes in integrated health services delivery more deeply, it is important to gain insight into the normative drivers behind the interactions between the actors in the network, and the relational contingencies, such as trust and goal consensus. This normative perspective may provide a better understanding of collaboration processes and the behaviors of actors, and thus insights into possible facilitating or hindering circumstances for effective network governance in different contexts.

The importance of the normative dimension of integration is also highlighted in conceptual frameworks on integrated care and integrated health services, developed to analyze their complexity. Fulop and colleagues [26] identify four levels of integrated care: organizational, functional, service and clinical integration. Organizational integration refers to the formal structure of the organization, functional integration to non-clinical support and back-office functions, service integration to how clinical services are offered and clinical integration to the process of care delivery to service-users. In addition to the different levels, the authors present two crucial dimensions of integration: systemic integration, which includes the coherence of rules and policies in the health system, and normative integration, which comprises the role of shared values in co-ordination and collaboration [26, 27]. Just as the conceptual model of Fulop and colleagues, the Rainbow Model of Integrated Care (RMIC), identifies four levels of integration: a system level, an organizational level, a professional level and a clinical level [20]. The RMIC also distinguishes two additional crucial dimensions: functional integration, referring to key support functions and activities, and again normative integration, which is defined as “the development and maintenance of a common frame of reference (i.e., shared mission, vision, values and culture) between organizations, professional groups and individuals” [20], (p., 8).

In addition to these conceptual frameworks, other integrated health services literature also mentions the role of values and normative integration. While some studies stress the importance of common values for cooperation in integrated health services delivery [28, 29], other research shows that the level of normative integration in integrated health services interventions in practice is still negligible [30]. Besides the attention for values and normative integration, there is a lack of information about what values are meant, and how they are defined. In the interim report ‘Global strategy on people-centred and integrated health services’ [8] the World Health Organization (WHO) stresses the need for a “unifying values framework” [8], (p., 11). The report defines a first set of guiding principles of integrated health services as ingredients for such a framework [8]. Therefore, although there is a desire to underpin normative integration and related behavior with a values framework, only a list of general principles has been compiled so far. Furthermore, this set has not been systematically assessed yet [31]. Thus, it is relevant to develop more scientific knowledge on the values underpinning the integrated health services concept, and what concrete values are meant.

As a first step towards more systematically developed knowledge on the values underpinning the integrated health services concept, a systematic review we conducted earlier identified a set of 23 underlying values of integrated care [32]. In that study we define values “as meaningful beliefs, principles or standards of behavior, referring to desirable goals that motivate action” [32], (p., 2). While this systematic review provides a balanced overview of values in the literature, it does not incorporate knowledge that has not been scientifically published. The set of values has also not been systematically empirically validated. Therefore, our next step is to systematically assess to what extent this initial set underpins the integrated health service delivery concept according to experts from multiple countries and professional perspectives, since integrated health services are delivered in a variety of contexts, settings and countries.

Besides identifying a first set of values underpinning the integrated care concept, our previous article also addresses that the application of these values might vary on the different levels of integration. This reflection is in line with the approaches of Fulop et al. (2005) and Valentijn et al. (2013), which assume that normative integration is a crucial dimension in determining how integrated health services delivery takes place on multiple levels, such as the personal, the professional, the management and the system level [20, 26]. However, not much knowledge about the relationship between values and levels of integration has been developed yet. Therefore, this study also investigates the relevance of the values on the different levels of integration.

The main research question of this study is: to what extent does the initial set of values underpin integrated care as a concept according to an international expert panel, and on what levels of integration are the values found to be relevant?

## Methods

To investigate to what extent the initial set of values underpins the concept of integrated health services delivery, and the relevance of the values on the levels of integration, we conducted an international Delphi study. A Delphi study is a systematic research method that uses the judgements of an expert panel, in order to reach consensus [33, 34]. The findings of our systematic review on values of integrated care served as the basis for the study [32]. As these findings did not include knowledge that has not been scientifically published, refinement by an international expert panel is an important next step before applying the values in further empirical research. A list of potential panel members was composed by tracking integrated health services publications and presenter lists of relevant conferences on health services research or integrated care. We aimed for a balanced expert panel, with a broad variety of expertise, professional disciplines and country backgrounds. In order to avoid bias, we excluded any of the first authors of the studies included in the systematic review [32]. Out of 65 invited experts, 33 (51%) agreed to participate in the Delphi study. Reasons for not participating were mainly limited time, leave or unavailability during one of the three Delphi rounds timeframes. The 33 experts originated from 13 different countries. The panel had an average age of 47 and an average of 11 years of experience in integrated health services. Panelists with a practice (30%), patient representative (6%), research (82%), policy (45%) and other (27%, e.g. education or advocacy) background participated in the study (see Table 1). Two experts were co-author in one of the studies included in the systematic review [32]. The expert panel members were asked to reflect on the set of values identified in the literature in three anonymous Delphi rounds. In every Delphi round, each expert received a personally generated hyperlink to an online questionnaire.

**Table 1:** Delphi expert panel characteristics (*n* = 33)

Characteristic	Category	Panel, *n* = 33
Age	Min-max	28–64
	Average	47
	Median	47
	SD	11
Gender	Male	36%
	Female	63%
Years of experience in integrated health services	Min-max	2–40
	Average	11
	Median	8
	SD	9
Background^a^	Practice	30%
	Patient representative	6%
	Research	82%
	Policy	45%
	Other	27%
Country	United Kingdom	6
	Australia	4
	Ireland	4
	Netherlands	4
	Canada	3
	Norway	3
	Belgium	2
	United States	2
	Austria	1
	Czech Republic	1
	New Zealand	1
	Spain	1
	Switzerland	1
Continent	Europe	70% (23)
	North America	15% (5)
	Oceania	15% (5)

^a^ = Multiple answers were possible

The panel members were asked to indicate for each value whether it underpins integrated care. To avoid central tendency bias, dichotomous answer categories (yes/no) were used at each question. The in- and exclusion criteria were as follows: in each round a value was included when a minimum of 80% of the panel members indicated it as underpinning, and excluded when a minimum of 50% of the panel members indicated it as not underpinning. These criteria were set based on methods used in comparable studies [35]. Values that were not included or excluded were presented again in the following round. Second, when assessing each value, the panel members had the opportunity to make a suggestion for reformulating the value and/or its description. All suggestions for reformulation were analyzed by the researcher, under the supervision of a second researcher. Minor suggestions, such as word order or replacement by synonyms (e.g. ‘service user’ instead of ‘client’), were implemented when they were suggested by multiple experts. Major suggestions listed by multiple experts, such as the addition of actors or activities in the description, were analyzed and presented to the expert panel in the next round.

Additionally, the panel members had the opportunity to suggest new values in rounds one and two. Suggestions for new values (including their descriptions) were analyzed by the researcher, under the supervision of a second researcher. If consensus could not be reached, a third researcher was consulted. New values and their description were presented to the panel in the next round.

Lastly, the relevance of the values on the levels of integration was investigated. When the panel members indicated a value as underpinning, they subsequently were asked on what level of integration the value is relevant. The response categories (multiple answers possible) were: ‘personal level’, ‘professional level’, ‘management level’ and ‘system level’, based on the RMIC [20]. The full Delphi questionnaire is provided in Supplementary file 1.

## Results

The Delphi study was conducted in three rounds. Delphi round one was completed by 33 experts. Two experts dropped out due to unexpected unavailability, resulting in a response rate of 94% in rounds two and three (see Table 2).

**Table 2:** Delphi study rounds overview

	Round 1	Round 2	Round 3
Response (*n* = 33)	100% (*n* = 33)	94% (*n* = 31)	94% (*n* = 31)
Values (*n*)	23	14	9
Included	12	5	1
Excluded	0	0	8
New	3	0	0

Eventually, 18 values were included in the refined set (see Table 3). In the first round, twelve values and descriptions were included: ‘co-ordinated’ (100%), ‘trustful’ (97%), ‘shared responsibility and accountability’ (94%), ‘holistic’ (94%), ‘co-produced’ (91%), ‘continuous’ (91%), ‘flexible’ (91%), ‘empowering’ (85%), ‘person-centered’ (85%, as a reformulation of ‘personal’), ‘respectful’ (85%), ‘led by whole-systems thinking’ (85%), and ‘comprehensive’ (82%). The expert panel included five values in round two: ‘collaborative’ (100%), ‘preventative’ (87%), ‘efficient’ (87%, newly suggested), ‘reciprocal’ (87%), and ‘transparently shared’ (80%, as a reformulation of ‘transparent’). In round three of the Delphi study, one value was included: ‘effective’ (90%, newly suggested). Two value labels were reformulated: ‘personal’ was reformulated in ‘person-centered’, and ‘transparent’ was reformulated into ‘transparently shared’.

**Table 3:** Delphi study results

#	Value label	Description
1	Co-ordinated	Connection and alignment between users, informal carers, professionals and organizations in the care chain, in order to reach a common focus matching the needs of the unique person.
2	Trustful	Enabling mutual trusting between users, informal carers, communities, professionals and organizations, in and across teams.
3	Shared responsibility and accountability	The acknowledgment that multiple actors are responsible and accountable for the quality and outcomes of care, based on collective ownership of actions, goals and objectives, between users, informal carers, professionals and providers.
4	Holistic	Putting users and informal carers in the centre of a service that is ‘whole person’ focused in terms of their physical, social, socio-economical, biomedical, psychological, spiritual and emotional needs.
5	Co-produced	Engaging users, informal carers and communities in the design, implementation and improvement of services, through partnerships, in collaboration with professionals and providers.
6	Continuous	Services that are consistent, coherent and connected, that address user’s needs across their life course.
7	Flexible	Care that is able to change quickly and effectively, to respond to the unique, evolving needs of users and informal carers, both in professional teams and organizations.
8	Empowering	Supporting people’s ability and responsibility to build on their strengths, make their own decisions and manage their own health, depending on their needs and capacities.
9	Person-centered^a^	Valuing people through establishing and maintaining personal contact and relationships, to ensure that services and communication are based on the unique situations of users and informal carers.
10	Respectful	Treating people with respect and dignity, being aware of their experiences, feelings, perceptions, culture and social circumstances.
11	Led by whole-systems thinking	Taking interrelatedness and interconnectedness into account, realizing changes in one part of the system can affect other parts.
12	Comprehensive	Users and informal carers are provided with a full range of care services and resources designed to meet their evolving needs and preferences.
13	Collaborative	Establishing and maintaining good (working) relationships between users, informal carers, professionals and organizations – by working together across sectors, and in networks, teams and communities.
14	Preventative	There is an emphasis on promoting health and wellbeing and avoiding crises with timely detection and action by and with users, informal carers and communities.
15	Efficient^b^	Using resources as wisely as possible and avoiding duplication.
16	Reciprocal	Care is based on interdependent relationships between users, informal carers, professionals and providers, and facilitates cooperative, mutual exchange of knowledge, information and other resources.
17	Transparently shared^a^	Transparently sharing of information, decisions, consequences and results, between users, informal carers, professionals, providers, commissioners, funders, policy-makers and the public.
18	Effective^b^	Ensuring that care is designed in such a way that outcomes serve health outcomes, costs, user experience and professional experience.

^a^ = value label has been reformulated

^b^ = value has been newly suggested in the Delphi study

**Fig. 1:**
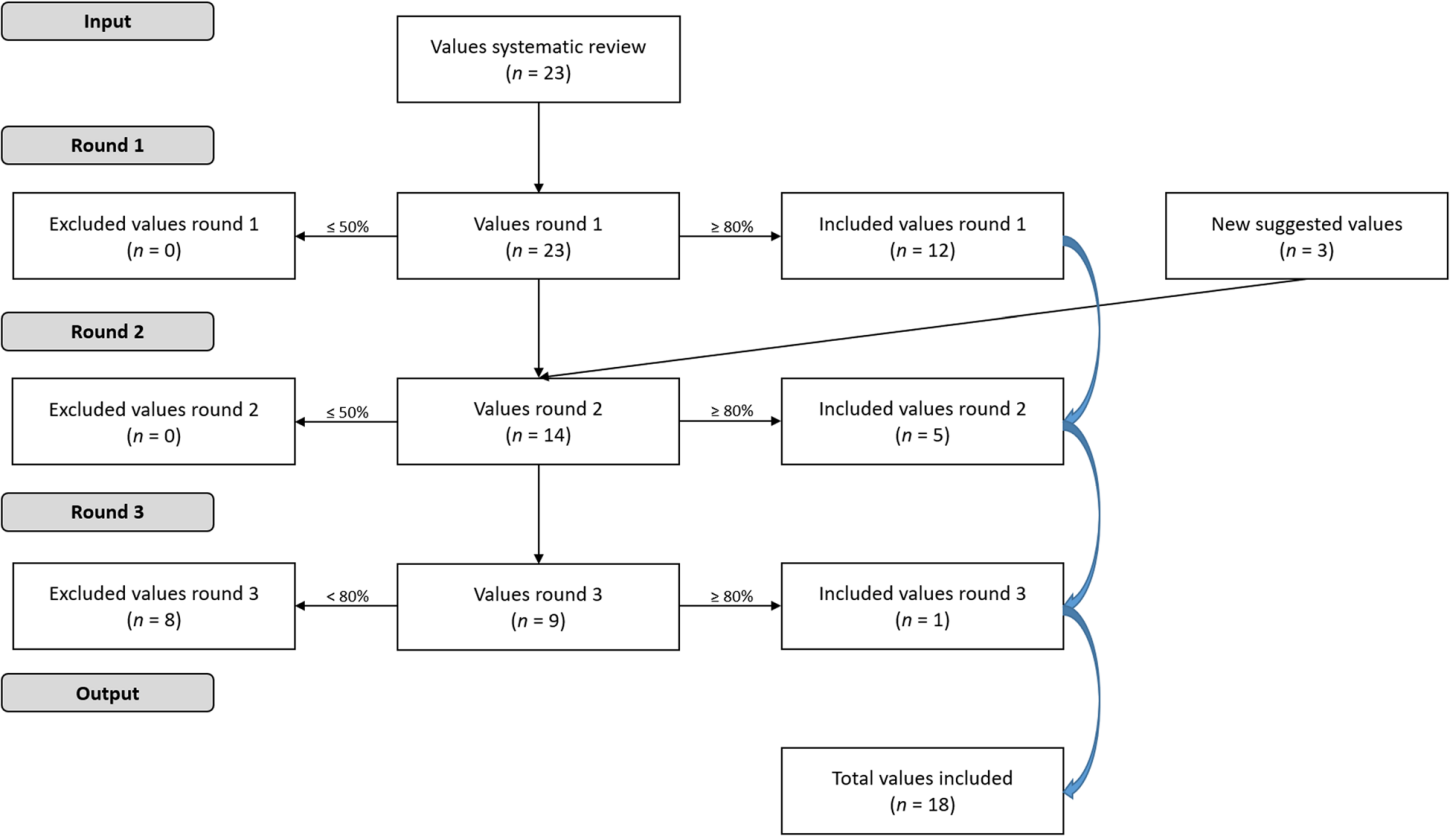
Delphi study flow chart Flow chart showing the three-round Delphi study process

In total, three new values were presented, all suggested in Delphi round one (see Table 2 and Fig. 1). The new values ‘effective’ and ‘efficient’ were suggested as a splitting of the value ‘sustainable’ of the initial set, which had both effective and efficient in its description (‘services are efficient, effective and economically viable, ensuring that they can adapt to evolving environments’). Furthermore, the new value ‘realistic’ was suggested and presented. Eventually, the new values ‘effective’ and ‘efficient’ were included in the refined set, and the value ‘realistic’ was excluded in the last round.

Eight values were excluded in round three of the Delphi study, due to not meeting the inclusion criteria. Seven of the excluded values were part of the initial set: ‘goal oriented’ (77%), ‘evidence-informed’ (73%), ‘equitable’(67%), ‘sustainable’ (73%), ‘innovative’ (67%), ‘proficient’ (63%), and ‘safe’ (73%). One of the values was newly suggested in round one: ‘realistic’ (73%). The main reasons for exclusion were: 1) the value is not specific enough for integrated care/integrated health services delivery (*n* = 8), and 2) the value is not essential for integrated health services delivery (*n* = 4).

### Levels of integration

In addition to studying to what extent the initial set of values underpins integrated care, the relevance of each value on the four levels of integration based on the RMIC was examined [20]: the personal, the professional, the management and the system level.

**Fig. 2:**
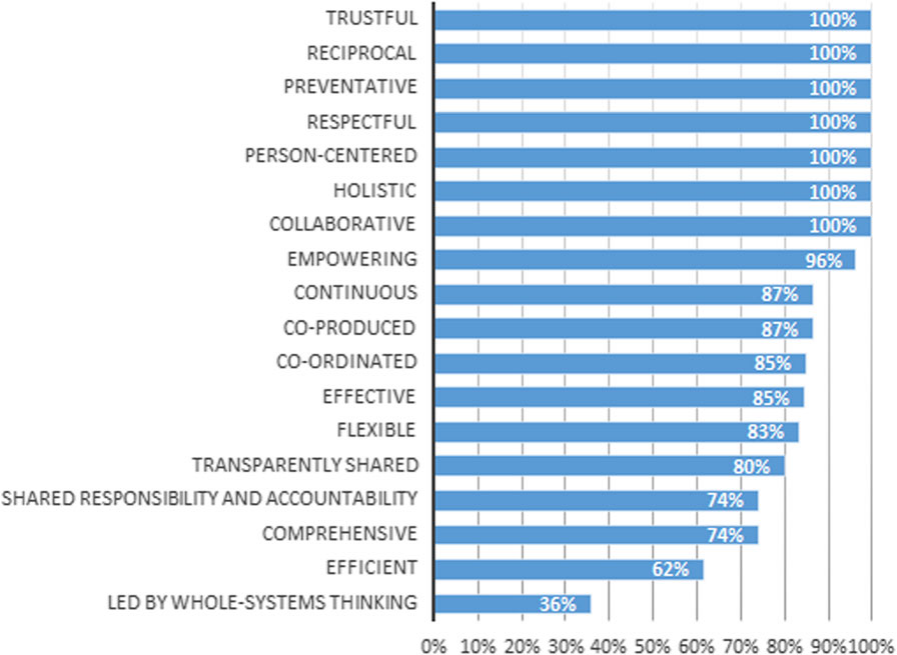
Average relevance scores of each value on the personal level Graph showing, for each value, the percentage of Delphi panel members that assessed the value as relevant on the personal level

**Fig. 3:**
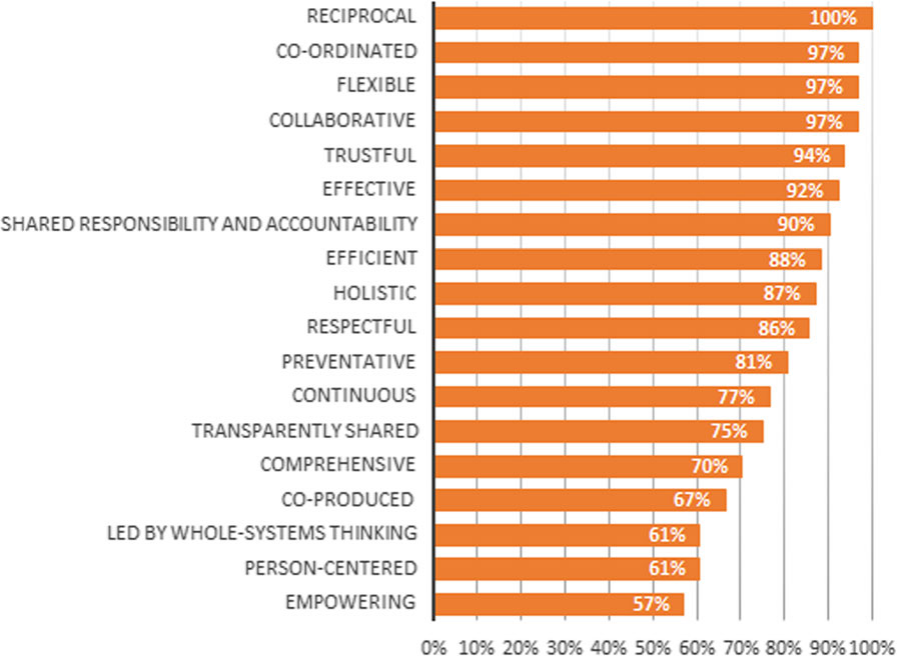
Average relevance scores per value on the professional level Graph showing, for each value, the percentage of Delphi panel members that assessed the value as relevant on the personal level

**Fig. 4:**
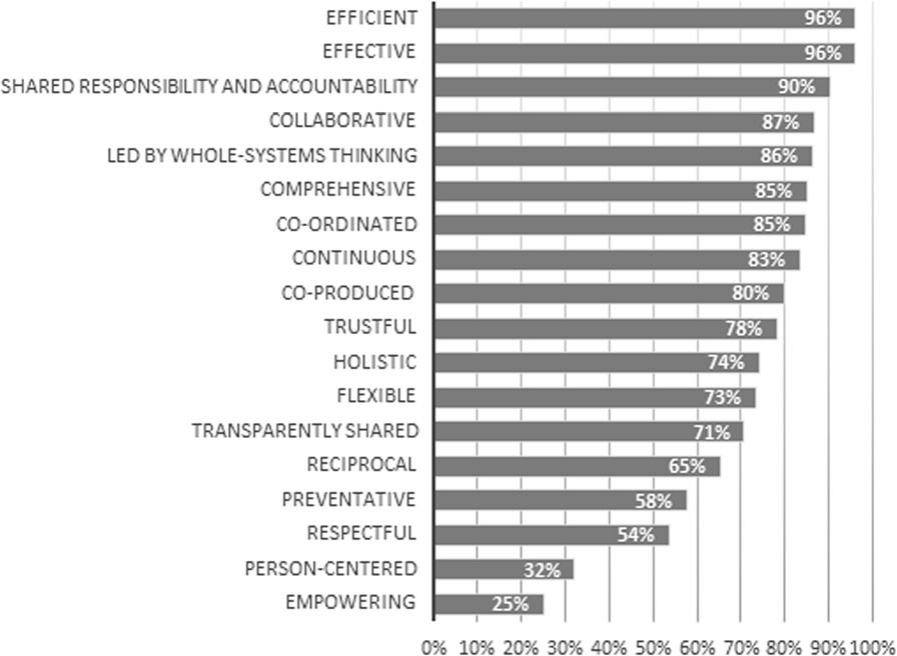
Average relevance scores of each value on the management level Graph showing, for each value, the percentage of Delphi panel members that assessed the value as relevant on the personal level

**Fig. 5:**
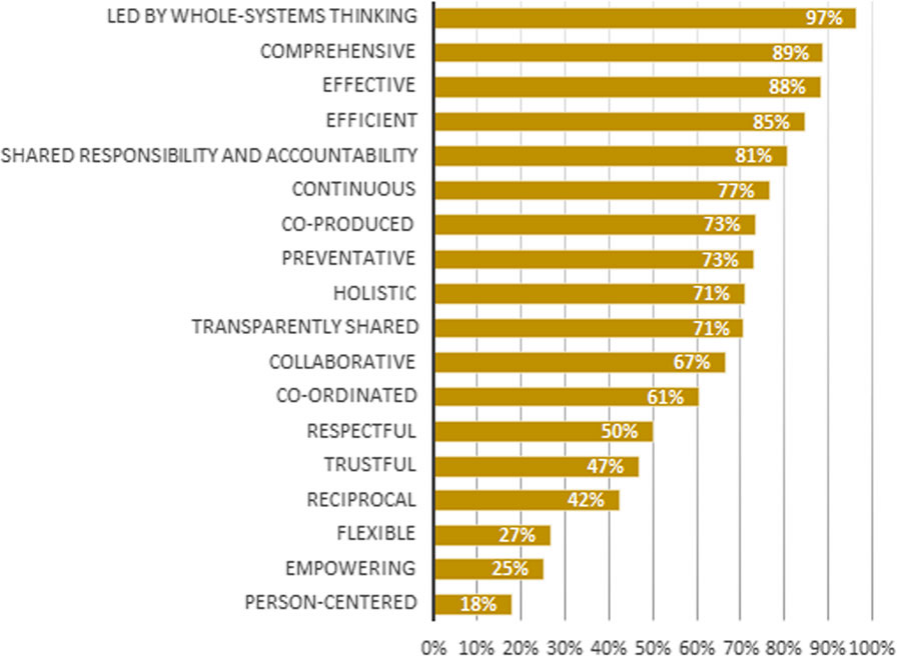
Average relevance scores of each value on the system level Graph showing, for each value, the percentage of Delphi panel members that assessed the value as relevant on the personal level

On the personal level (see Fig. 2), the values ‘trustful’, ‘reciprocal’, ‘preventative’, ‘respectful’, ‘person-centered’, ‘holistic’ and ‘collaborative’ achieved 100% relevance scores. This means that each panel member found these values relevant on the personal level. The values ‘led by whole-systems thinking’ (36%) and ‘efficient’ (62%) were assessed as least relevant on the personal level. The values with the highest relevance scores on the professional level (see Fig. 3) are ‘reciprocal’ (100%), ‘co-ordinated’ (97%), ‘flexible’ (97%), ‘collaborative’ (97%), ‘trustful’ (94%), ‘effective’ (92%) and ‘shared responsibility and accountability’ (90%). ‘Empowering’ (57%), ‘led by whole-systems thinking’ (61%) and ‘person-centered’ (61%) were assessed as least relevant on the professional level by the expert panel. When looking at the management level, the values ‘efficient’ (96%), ‘effective’ (96%) and ‘shared responsibility and accountability’ (90%) were assessed as the most relevant (see Fig. 4). The values with the lowest relevance scores are ‘empowering’ (25%), ‘person-centered’ (32%), ‘respectful’ (54%) and ‘preventative’ (58%). Lastly, on the system level (see Fig. 5) ‘led by whole-systems thinking’ (97%), ‘comprehensive’ (89%), ‘effective’ (88%) and ‘efficient’ (85%) are assessed as the most relevant values. The lowest scoring values on the system level are ‘person-centered’ (18%), ‘empowering’ (25%), ‘flexible’ (27%), ‘reciprocal’ (42%) and ‘respectful’ (47%).

**Fig. 6:**
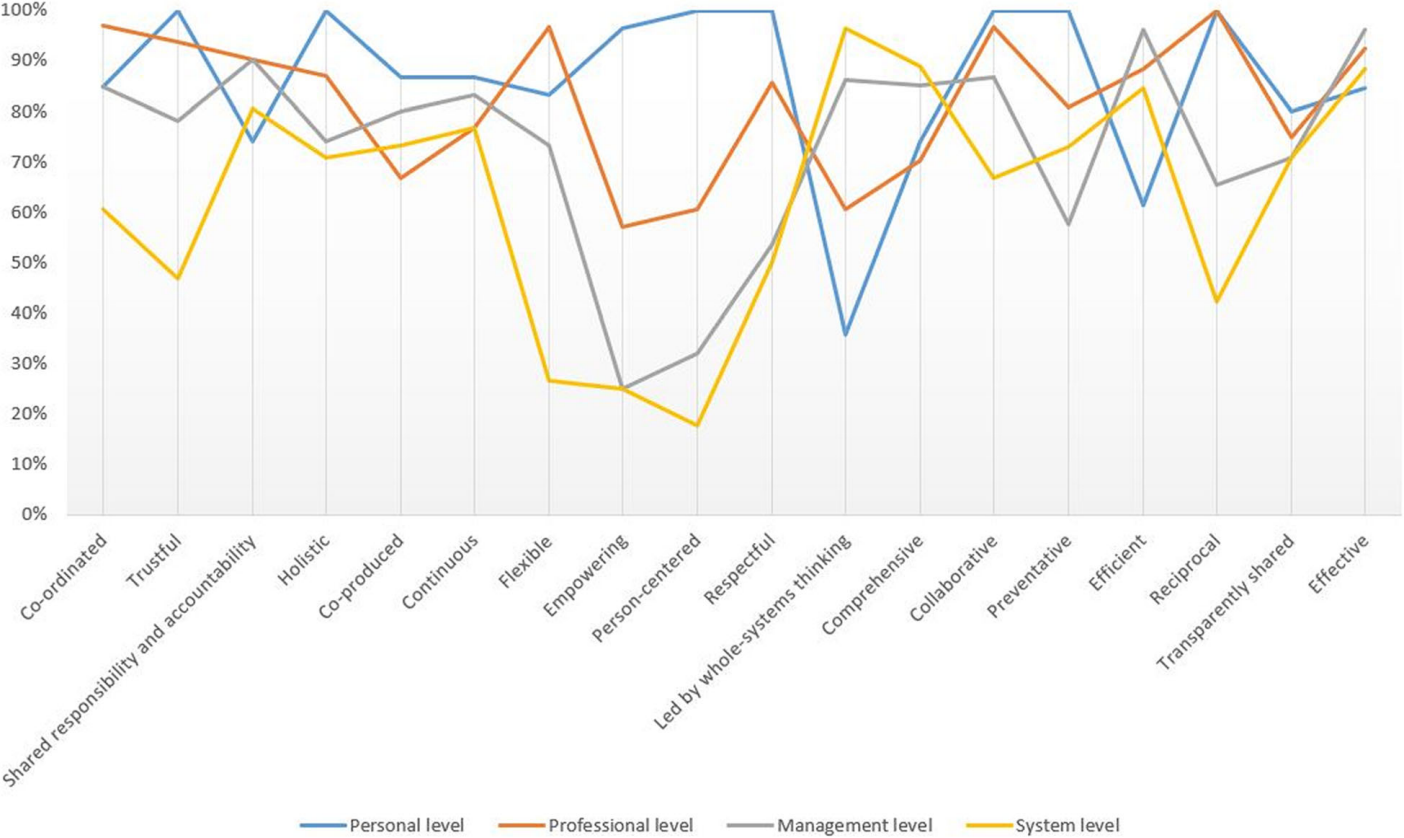
Average relevance scores of each value on each level Graph showing, for each value, the percentage of Delphi panel members that assessed the value as relevant on each of the levels of integration

Furthermore, differences can be seen between the relevance scores of each value on each level of integration. Some of the values seem to be highly relevant at multiple levels of integration. For example, the value ‘effective’ scores respectively 85, 92, 96 and 88% on the personal, professional, organizational and system level. The relevance scores of other values are less equally distributed among the levels of integration. For example, the value ‘person-centered’ shows relevance scores of respectively 100, 61, 32 and 18% on the personal, professional, organizational and system level. Figure 6 presents the relevance scores of the values on each level.

## Discussion

The aim of this study was to develop elements for a conceptual values framework for integrated health services delivery, which contributes to our understanding of the normative aspects of integrated health services delivery. Our study refined and validated an initial set of values based on the literature. Furthermore, the relevance of the values on the levels of integration as defined by the RMIC was studied [20]. The refined values set consists of 18 values of integrated health services, including a value label, a description of each value, and a relevance score on each level of integration. Of the initial set of 23 underlying values of integrated care, 16 values (70%) were included in the final set. Two value labels were reformulated and two new values were added. Eight values of the initial set were excluded by the expert panel because they were assessed as not specific or essential enough for integrated care/integrated health services delivery. The study resulted in an international normative basis for the concept of integrated health services delivery. While context, developments and interventions in integrated health services delivery may vary between and within countries, the study demonstrated that consensus can be reached about what values underpin integrated health services delivery or integrated care as a concept. By using the expertise of 33 experts from 13 different countries and multiple professional backgrounds, the developed set of values has a broad base. The results also demonstrate that the literature-based systematic review [32] provided a strong basis for the initial set, because the number of new values was limited and the added elements were partly present in the values of the initial set. The knowledge of the international experts provided additional insights for refinement.

The findings of this study also provide insight into which values are relevant on which levels of integration. On the personal level, values closely related to the interaction with service users are found to be most relevant. Examples are ‘holistic’, ‘trustful’, ‘respectful’ and ‘empowering’. This corresponds to studies identifying core components of person-centered care that also recognize these dimensions [36–38]. When looking at the most relevant values on the professional level, such as ‘collaborative’, ‘co-ordinated’, ‘reciprocal’ and again ‘trustful’, they mostly relate to collaboration between professionals. These values are also found to be relevant in the literature that analyzes interprofessional collaboration as a concept [21, 39, 40]. On the management level, the highest scoring values ‘effective’, ‘efficient’ and ‘shared responsibility and accountability’ are correspondingly reflected in articles that approach healthcare delivery from a business or quality management approach e.g. the application of LEAN management [41, 42]. Lastly, the values that are identified as most relevant on the system level, like ‘led by whole-systems thinking’ and ‘comprehensive’, are also reflected in reports that describe strategic directions for health systems design [8, 12]. Thus, the relevance scores of the values on the different levels of integration are underpinned by the existing literature. Moreover, these findings seem to demonstrate that the most relevant values on the personal and professional levels relate to interpersonal aspects, while the most relevant values on the management and system levels are associated with rational aspects.

Furthermore, this study illustrates that some of the values, like ‘effective’ (85, 92, 96, 88%), score consistent across the different levels of integration, while other values, like ‘empowering’ (96, 57, 25, 25%), score inconsistent across these levels. The consistent scores, on the one hand, may provide insight into the interconnectedness of values across different levels of integration. For example, supporting holistic ways of working on the professional level (e.g. multidisciplinary teams) facilitates the delivery of holistic care on a personal level. Vice versa, non-holistically organized funding streams or sector specific legislation may complicate the delivery of holistic health services on the micro level. Moreover, when for instance striving for efficiency on a system level, it is likely efficiency-driven incentives are present in the relationships between service users and professionals. While these consistent scores indicate that the 18 values are connected across the different levels of integration, the more divided or inconsistent scores, on the other hand, suggest that there are also certain differences in emphasis on the values on these levels. A value like ‘empowering’ may, for instance, be more relevant on the personal level than on the system level. These insights suggest that it is important to consider the interconnectedness of values on multiple levels of integration in integrated health services networks, including particular differences in emphasis per level. When applying the values framework in practice, it is thus important to be aware on what level of integration you are operating. On some levels certain values could be more or less relevant.

When looking at the results from a values theory point of view, the 18 values presented appear to be instrumental values and might be underpinned by certain terminal values. Value theorists such as Rokeach, Schwartz and Bilsky [43–45] distinguish two categories of values: 1) values that represent terminal goals (end states), and 2) values that represent instrumental goals (modes of behavior). Terminal values are phrased as nouns, for instance ‘freedom’ or ‘security’, while instrumental values or phrased as adjectives such as ‘free’ or ‘secure’. So, terminal values are end goals, whereas instrumental values represent the process by which these goals are achieved. Because integrated health services delivery can be considered as a process [12], we have chosen to formulate a set of instrumental values underpinning the concept of integrated health services delivery. The 18 values presented describe certain modes of behavior (instrumental goals). For example ‘empowering’, which refers to the process of supporting people’s ability and responsibility. Furthermore, all values are phrased as adjectives e.g. ‘holistic’ and ‘comprehensive’. On the other hand, considering the insights of Rokeach, Schwartz and Bilsky, it is likely that there are certain terminal values that underlie the 18 instrumental values of integrated health services delivery. Examples of these terminal values could be ‘self-determination’, ‘freedom’ or ‘a healthy life’. Because these terminal values represent desirable end states, they may help describe impact and end goals. Terminal values could therefore play an important role in defining quality of services, impact on service users and informal carers, and objectives of integrated health services programs. It would be relevant to further investigate the dichotomy between terminal and instrumental values, and its practice implications.

Additionally, it is relevant to consider that the 18 values presented are determined by many factors. Although this study strongly focused on the identification of values underpinning the concept of integrated health services delivery, values are influenced by many factors. In addition to personal determinants such as age, gender and family characteristics, there are also socio-cultural influences like education, previous experiences, occupation and culture [43, 46]. On the one hand, personal values can influence work behavior. For example, studies report on relationships between the personal values of employees and their decision-making styles [47], their ethical behavior [48] and their attitudes [49]. On the other hand, individuals also internalize professional and organizational values through socialization. This is described by studies that identified common professional values of nurses [50, 51] and value systems of organizations [52]. Therefore, when using the presented set of 18 values, it is important to be aware that this set is a result of an interplay of individual, professional and organizational values. Since integrated health services delivery is an interorganizational undertaking, contrasting organizational values may complicate collaboration in networks.

By providing insight into the normative aspects of integrated health services, the presented set of values can also contribute to the understanding of its governance. Since integrated health services delivery is a multidimensional undertaking that transcends organizations, new governance mechanisms and instruments are needed [23]. These new governance mechanisms should connect organizations, sectors and people. Values may play an important role in this, since that behavior, interaction and decision-making in integrated health services networks are strongly influenced by the values of the stakeholders involved [46]. However, those values lie underneath these processes and are not often made explicit. The set of values provides a vocabulary and framework for making the values of the stakeholders in the network more explicit. In this way, the underlying mechanisms of integrated health services networks can be understood more deeply. Similarities and differences in the value priorities of the stakeholders, known as value hierarchies [43, 46, 53], can be uncovered. Different interpretations of values can also be identified. For example, the meaning of a value like ‘person-centeredness’ may be different for individuals from different professional backgrounds. Explication of the value priorities and interpretations of the stakeholders provides insight into how the governance of integrated health services networks can be organized, and what the possible enabling circumstances or barriers are. On the one hand, a set of shared values and meanings might enable the development of common ground [24], mutual understanding and shared motivation [54]. All of these are known as important factors or contingencies for the organization of shared governance [24, 25, 54]. On the other hand, clarification of the differences between the individual value priorities offers insights into possible barriers, and may not necessarily affect trust or goal consensus in the network. When no shared governance values can be agreed upon, other network governance forms such as the centralized ‘lead organization governance’, might also be considered [25]. Value congruence might therefore form an additional network contingency.

### Practice implications

In order to understand and organize shared governance in integrated health services delivery, it is important to gain insight into the values of the different stakeholders in the network. Although this study presents a comprehensive framework of values underpinning the concept of integrated health services delivery, people may have different value priorities and interpretations. The values of service users, informal carers, professionals, managers or policymakers may sometimes even conflict. It is therefore relevant to be aware of the values and possible value conflicts in integrated health services and how to deal with those conflicts. In practice, the set of values can be used as a vocabulary tool to make values more tangible and explicit. It is important to start a fundamental discussion about which values are the most important for each stakeholder, what their meaning is, and what values are being missed in the current situation of the network. The most important values can be identified by prioritizing. Subsequently, similarities and differences in the value priorities and interpretations of the stakeholders can be uncovered, and the most important collective values can be identified. Additionally, the values that are seen as most important or the values with the least consensus, can be discussed more thoroughly by collectively giving meaning to them. This is important because people from different backgrounds and disciplines often have a different interpretation of values. This overview can be used to further align collaboration, governance and decision-making. Common collective values could be used as a shared point of departure for the further development of integrated health services networks. A set of leading values could, for instance, form the basis for the future strategy and policy. On the other hand, as organizations and networks are made up of people, conflicting values may also exist. Discussing these values can help to find mutual understanding and common ground. It could provide understanding of underlying drivers, views and interests.

Furthermore, the values can contribute to the evaluation of performance and the guidance of behavior in integrated health services. Because the values refer to desirable situations, they can form a basis for the evaluation of performance and quality on the four levels of integration [20, 26]. When an integrated health service identifies ‘respectful’ as leading value, this could be monitored by measures related to respectfully delivering health services. On the personal and professional level, values could be incorporated in the service user, informal carer and employee satisfaction surveys. On the management and system level, values could be developed into indicators which can be monitored and supervised over time. Correspondingly, values can form a frame of reference for individuals in daily work and decision-making. When, for example, a value as ‘empowering’ is identified as a leading value in an integrated health service, professional teams should continuously consider whether service users can make their own decisions in every activity or action we carry out. In this example, values provide a framework for professionals to make decisions based on a value consideration. This could make them more accountable for their decisions.

In conclusion, values can play an important role in the total package of governance functions in integrated health services: leadership, supervision and accountability [23]. First, values can play a role in leadership by forming a backbone for determining the objectives, mission and vision of an integrated health service. Shared and conflicting values could also form a vocabulary for determining ethics and creating culture in integrated health services. Furthermore, values can form the basis for supervision and accountability functions. First, by providing both a basis for measures which can be supervised over time. Second, by providing a framework for daily practice which could make people more accountable.

### Further research

The set of 18 values presented forms a basis for empirical research in integrated health services delivery. For example, it would be valuable to further empirically examine how the values relate to the actual practical behavior and actions of people in integrated health services delivery, within and between organizations [46, 47]. Considering the insights of value theorists as Schwartz, Rokeach, Hitlin and Piliavin [43, 46, 53], values transcend specific situations. As they are not uniform, they can be interpreted and applied differently in different contexts. It could be relevant to gain more insight into these different appearances of values and their relationship to contextual factors. For instance, to study which values are specifically relevant in decision-making processes, and to what extent these values can be recognized in behavior and actions of stakeholders. This could be investigated in empirical case studies. Furthermore, it would be relevant to further study the relationship between the normative aspects of integration and the organization of governance in the network. For example, to examine the dynamics between organizational and network values, to investigate to what extent values need to be shared, in order to effectively govern integrated health services, or to study how normative integration relates to the creation of a mutual understanding or trust [22–25, 54]. Another direction for further research may be the examination of differences and similarities concerning the relative value priorities between stakeholder groups (beyond ‘experts’) in integrated health services delivery. Since values are determined by both personal and socio-cultural factors [43, 46], differences between stakeholder groups (e.g. service users, informal carers, professionals, policymakers, managers) or geographical differences may appear. For the understanding of integrated health systems, it would also be valuable to gain insight into how value differences and contradictions on the different levels of integration influence one another and how this affects outcomes such as employee satisfaction or the effectiveness of the system.

### Strengths and limitations

A strength of this study is the basis of a systematic review as a starting point, enriched by expert knowledge. The Delphi panel included 33 international experts with a large experience from 13 different countries in the field integrated health services delivery. Only two experts dropped out, resulting in a 94% response rate. The experts also reached a satisfactory convergence of opinion and saturation after three Delphi rounds, whereas no more new values were suggested. Another strength of this study is its innovative nature and contribution to the existing body of knowledge. Although the relevance of normative integration is confirmed in the literature [20, 26–29], the WHO stresses the need for a values framework [8], and professional and governance codes plead for values-driven approach [55, 56], no systematically assessed values set from a multi-organizational perspective was developed yet. This study adds to this gap in knowledge. By delivering ingredients for a values framework for the concept of integrated health services delivery, this study fills a gap in knowledge.

Our study also has its limitations. Although an international panel with high expertise was involved, the number of experts per country was limited. Some countries (Austria, Czech Republic, New Zealand, Spain and Switzerland) were, for instance, represented by only one expert. Another limitation is that the participating experts all originated from Western countries, which makes it difficult to draw any conclusions on a global scale. It would therefore be valuable to also validate the values framework in Africa, Asia, South America and low and middle income countries. Furthermore, the questionnaire was only available in the English language. Although all panel members had a good understanding of the English language, not every expert had English as a native language. This could have led to different interpretations. Lastly, most of the participating experts had a background in research, policy or practice while the values of other stakeholders in integrated health services delivery (such as service users and informal carers) may be different. The examination of the service user or informal carer perspective on the developed values set is therefore an important suggestion for further research.

## Conclusions

In order to organize health services delivery in a less fragmented and a more person-centered way, it is important to integrate health services. To align collaboration and shared governance in integrated health services networks efficiently, a deeper understanding of the normative dimension of health services integration is necessary. In addition to functional aspects such as activities and interventions, the values that drive the actors’ behavior play a role in collaboration. Therefore, more knowledge on what values underpin the integrated health services concept is needed. This study systematically investigated to what extent an initial set of underlying values derived from literature underpins integrated health services by conducting an international Delphi study with 33 experts from 13 countries. Additionally, the relevance of the values on the levels of integration was studied. This resulted in ingredients for a values framework for integrated health services, consisting of 18 values and descriptions, including a relevance score on the levels of integration: personal level, professional level, management level and system level. The set of values forms an international normative basis for integrated health services delivery. It delivers ingredients for a framework that could form a basis for a better understanding of the normative dimension of integration and the dynamics in shared governance processes in integrated health services delivery networks.

## Supplementary information

**Additional file 1.** Questionnaire Delphi study File showing the full three-round Delphi online questionnaire

## Data Availability

The datasets generated and/or analyzed during the current study are not publicly available due because the confidentiality and privacy of the expert panel members is respected.

## Abbreviations

RMIC: Rainbow Model of Integrated Care
WHO: World Health Organization
