# Supplementary material for: Towards a values framework for integrated health services: an international Delphi study
Source: BMC Health Serv Res. 2020 Mar 18;20:224. doi: 10.1186/s12913-020-5008-y (PMC7079447; doi:10.1186/s12913-020-5008-y)
Supplement: Supplementary file 1 — Additional file 1. Questionnaire Delphi study File showing the full three-round Delphi online questionnaire [file 12913_2020_5008_MOESM1_ESM.pdf]

Delphi study 1 design - a set of values underpinning integrated care

Delphi Study Round 1

Input:	#	Question	Answer options	Decision	
List of values resulting from **literature review**		*Per value:*			
	1	Does this value underpin integrated care?	No	> 50%	Exclusion
			Yes	> 80%	Inclusion
			> Explanation	Remaining values	Round 2
	2	Suggestions for possible rephrase	[Open]	Analysis	Round 2
	3	When 'yes' at Q1: On which is this value relevant (multiple answers possible)?	Personal level		Stats
			Professional level		Stats
			Management level		Stats
			System level		Stats
		Closing question:			
	4	Would you like to add any value(s) that may be missing in this list?	[Open]	Analysis	Round 2

Delphi Study Round 2

Input:	#	Question	Answer options	Decision	
List of values resulting from **Delphi round 1**		*Per value:*			
	1	Does this value underpin integrated care?	No	> 50%	Exclusion
			Yes	> 80%	Inclusion
				Remaining values	Round 3
	2	Suggestions for possible rephrase	[Open]	Analysis by research team and possible rephrasing	Round 3
	3	When 'yes' at Q1: On which is this value relevant (multiple answers possible)?	Personal level		Stats
			Professional level		Stats
			Management level		Stats
			System level		Stats
		Closing question:			
	4	Would you like to add any value(s) that may be missing in this list?	[Open]	Analysis by research team and possible addition to list	Round 3

Delphi Study Round 3

Input:	#	Question	Answer options	Decision	
List of values resulting from **Delphi round 2**		*Per value:*			
	1	Does this value underpin integrated care?	No	> 50%	Exclusion
			Yes	> 80%	Inclusion
			> Explanation	Remaining values	Exclusion
	2	Suggestions for possible rephrase	[Open]	Analysis	Def.list

	3	When 'yes' at Q1: On which is this value relevant (multiple answers possible)?	Personal level			Stats
			Professional level			Stats
			Management level			Stats
			System level			Stats
	Closing question:					
	4	Would you like to add any value(s) that may be missing in this list?	[Open]	Analysis		Def.list
